# A Case of Self-Detachment of a Stage 3 Idiopathic Epiretinal Membrane Due to Posterior Vitreous Detachment

**DOI:** 10.7759/cureus.95850

**Published:** 2025-10-31

**Authors:** Chiaki Matsui, Natsuki Ueji, Yoko Mase, Akiko Kubo, Mineo Kondo

**Affiliations:** 1 Ophthalmology, Kinan Hospital, Mie, JPN; 2 Department of Ophthalmology, Mie University Graduate School of Medicine, Tsu, JPN

**Keywords:** epiretinal membranes, optical coherence tomography (oct), posterior vitreous detachment (pvd), self resolution, surgery start time

## Abstract

We report our findings in a case in which the retinal traction caused by an epiretinal membrane (ERM) was relieved by a spontaneous posterior vitreous detachment (PVD). A 53-year-old woman complained of visual disturbances in both eyes. Her decimal best-corrected visual acuity (BCVA) was 1.2 (about 20/16 in Snellen units) in both eyes with no abnormalities in the anterior chamber, lens, or vitreous. Ophthalmoscopy and optical coherence tomography revealed a Stage 3 ERM in the right eye with a foveal macular thickness (FMT) of 408 μm and an intact posterior hyaloid membrane. The M-chart score was 0° in both the horizontal and vertical directions. She was followed with observation only. Eight months later, the patient returned with a complaint of floaters in the right eye. Her decimal BCVA remained at 1.2 in both eyes with no retinal tears, retinal detachment, or vitreous hemorrhage. However, a PVD was detected with a detachment of the ERM into the vitreous cavity. The FMT had decreased to 389 μm, and the macular traction appeared to be relieved. The detachment of the ERM reduced the retinal traction, and the BCVA was maintained during the 27-month follow-up period. This case shows the long-term stability without decreasing BCVA or recurrence of an ERM. We suggest that the detachment of the ERM was due to the development of a PVD which then led to the release of retinal traction. We recommend that surgery not be performed in cases of idiopathic ERM without a PVD and with good visual acuity and no metamorphopsia. Instead, these cases should be carefully monitored for visual symptoms and a worsening of the traction on the retina.

## Introduction

An idiopathic epiretinal membrane (ERM) is an essential cause of visual impairments because of its tractional effects on the retinal structure (collectively known as ‘macular pucker’). It typically develops in individuals > 50 years of age, although it can occur in association with a number of retinal diseases. It can also occur in isolation. The formation of an ERM is characterized by a number of pathological changes occurring in the vitreoretinal junction, and it can occur after a partial or complete posterior vitreous detachment (PVD) in 80-95% of cases [1-5].

Clinically, an ERM can cause a decrease in visual acuity and metamorphopsia, and a surgical intervention such as membrane peeling during vitrectomy is one of the options to treat eyes at the advanced stages of ERMs. Although a spontaneous separation of an idiopathic ERM from the retinal surface has been reported, it occurs in only 1% to 3% of all ERM cases [1-4,6-9].

We report our findings in a case of a spontaneous release of traction on the retina due to the detachment of an ERM from the internal limiting membrane (ILM) that was associated with a PVD. This case suggests that surgical treatment for an ERM can be delayed and careful monitoring for signs of a worsening of the traction on the retina be performed.

## Case presentation

**Figure 1:**
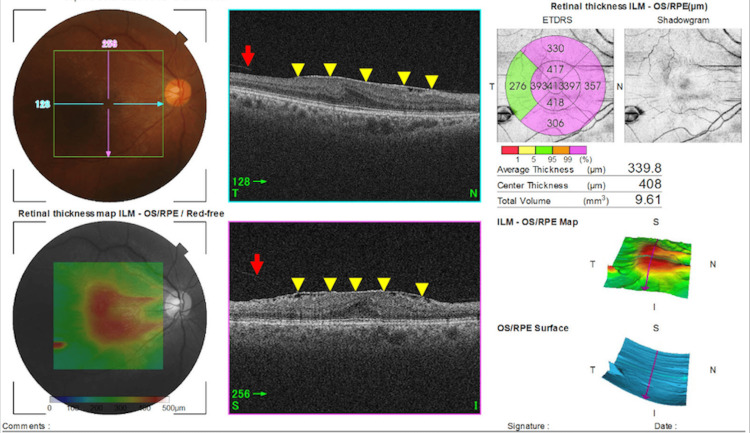
Optical coherence tomographic (OCT) images of the right eye showing a Stage 3 epiretinal membrane (ERM). Swept source-OCT (Topcon) at the time of the initial diagnosis showing a stage 3 ERM (yellow arrowheads) and an intact posterior hyaloid membrane (red arrow).

A 53-year-old woman presented with complaints of double vision when both eyes were open. Our examinations showed that her decimal best-corrected visual acuity (BCVA) was 1.2 (about 20/16 in Snellen units) in both eyes. She had binocular diplopia with an 8Δ of intermittent exotropia for near vision, and a 4Δ of intermittent exotropia for far vision. Slit-lamp examination showed no abnormalities in the anterior chamber, lens, or vitreous. The swept source optical coherence tomographic (OCT; Topcon, Tokyo, Japan) images showed that the ERM was a Stage 3 ERM (Figure 1) in the right eye. The foveal macular thickness (FMT) was 408 μm, and an intact posterior hyaloid membrane was present. She did not have monocular diplopia. The M-chart score was 0° in both the horizontal and vertical directions. The left eye had no abnormalities. 

The FMT was greater than the normal range [10], and a retinal traction by the ERM was suspected to be the cause of the increase in the FMT. We concluded that the diplopia was binocular diplopia due to the intermittent exotropia. Because of her good visual acuity, absence of metamorphopsia, and an intact ellipsoid zone (EZ) as confirmed by OCT volume scan images, she was followed without surgery. 

**Figure 2:**
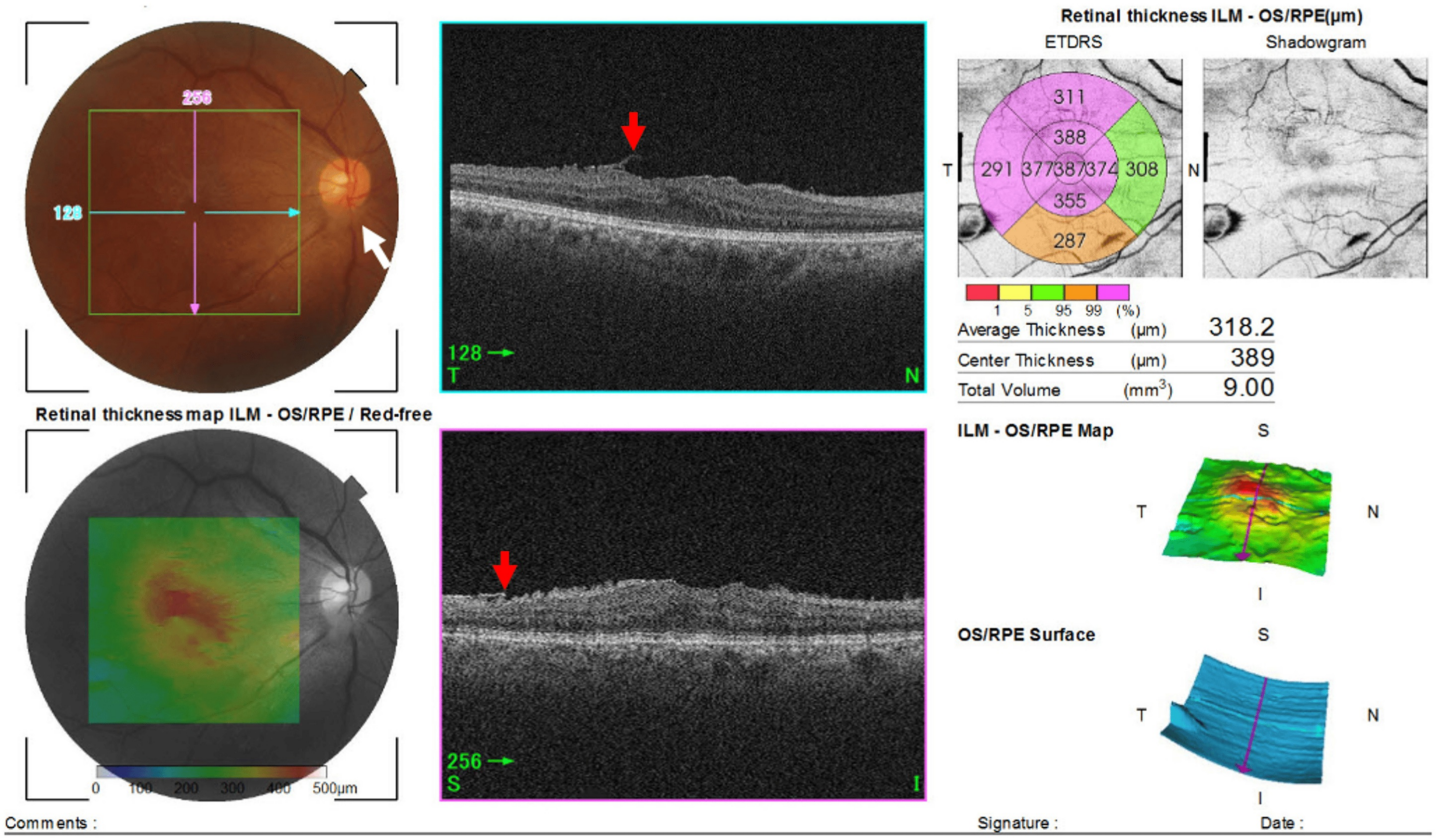
Optical coherence tomographic (OCT) image of the right eye showing a detachment of the epiretinal membrane (ERM) and a posterior vitreous detachment (PVD). Swept source-OCT (Topcon) eight months after the ERM diagnosis shows a detachment of the ERM (red arrow), foveal macular thickness (FMT) reduction, and a posterior vitreous detachment. A Weiss ring (white arrow) can also be seen.

Eight months later, the patient returned with a complaint of floaters in the right eye. Her decimal BCVA remained at 1.2 in both eyes with no retinal tear, retinal detachment, or vitreous hemorrhage. However, a PVD was confirmed with the detachment of the ERM from the retina. The flap was seen floating in the vitreous cavity (Figure 2). The FMT was reduced to 389 μm, and the macular traction was weakened as confirmed by comparing the thickness maps in Figures 1, 2.

**Figure 3:**
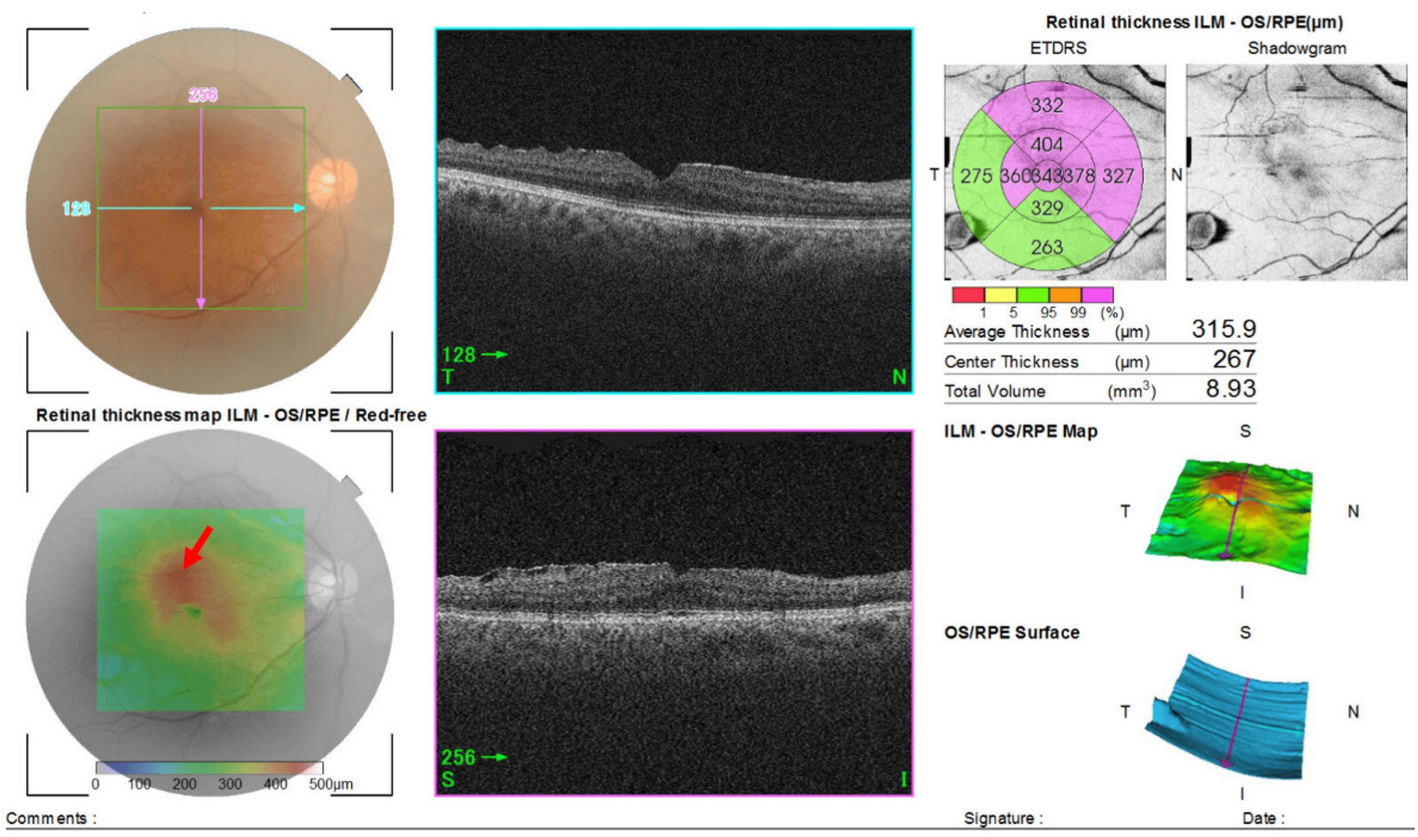
Optical coherence tomographic (OCT) image of the right eye showing the changes of retinal traction by the epiretinal membrane (ERM). Swept source-OCT (Topcon) 11 months after the ERM diagnosis showing a reduced foveal macular thickness (FMT) due to the release of macular traction at the area of the detached ERM (red arrow). However, the retinal traction at the area of the residual ERM showed a slight progression.

Our examination three months later showed that she still had floaters, and the decimal BCVA remained stable at 1.2 in both eyes with no retinal tears, retinal detachment, or vitreous hemorrhage. The FMT was reduced to 267 μm, however the retinal traction at the area of the residual ERM showed signs of a slight increase (Figure 3). We selected to continue to follow this patient without any surgical intervention.

After 27 months of follow-up examinations, her BCVA had remained stable and a recurrence of an ERM was not found.

## Discussion

A spontaneous detachment of an ERM is not a common occurrence [1-4,6,7], and it has been reported to occur in approximately 1% to 3% of all ERM cases [6]. A spontaneous detachment of a full-thickness, free, or partial flap ERMs, has been observed in cases of secondary ERMs especially in adolescents, and in cases of ERMs that developed after inflammatory retinal diseases [3,8,9]. In contrast, a spontaneous detachment of an idiopathic ERM from the retinal surface is a rare occurrence [1-4,6,7]. Previous studies have suggested that the mechanism by which ERM self-detaches is caused by the ERM being pulled away by a PVD of the vitreous body. In these cases, the anteroposterior tractional forces of a PVD overcome the tangential tractional forces, and the base of the already thin ERM detaches from the retina in the form of a flap in the periphery [1,3,7,11].

Another suggestion for a spontaneous detachment is a tangential traction of the ILM caused by the contraction of the ERM is stronger than the adhesion of the ERM to the retina. This would then lead to a separation of the ERM from the retina [1,7,11]. 

Another possibility is that the weakest structural part of an ERM breaks by stress such as a severe and acute increase of the intraocular pressure resulting in contraction toward the epicenter. Mansour et al. [12] reported a spontaneous separation of an ERM in a young weight-lifting athlete after heavy weightlifting with a sensation of severe eye pressure.

Under this condition, the mechanism associated with a PVD was suspected.

**Figure 4:**
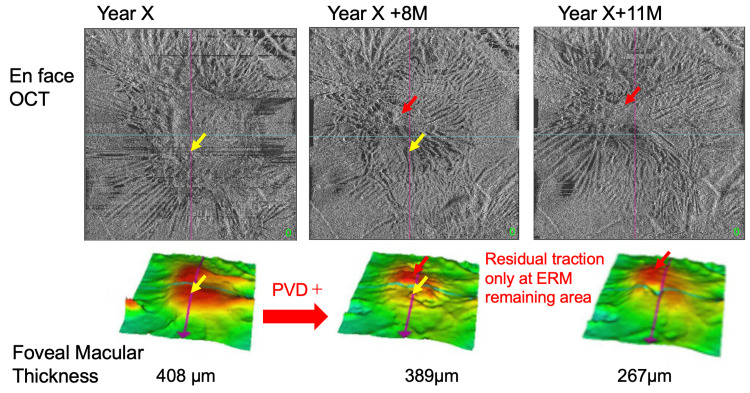
En face optical coherence tomographic (OCT) images of the right eye showing a decrease in the foveal macular thickness (FMT) due to a relief of the macular traction (yellow arrow) and slight progression of the retinal traction at the area with residual epiretinal membrane (ERM) (red arrow).

Enface OCT images showed the changes in the foveal macular thickness in our case (Figure 4). The traction on the central fovea was relieved by the PVD. However, in the area where the ERM remained attached to the internal limiting membrane, a localized increase in the traction of the retina was observed (see red arrow in Figure 4) even after the PVD had developed. Earlier studies have reported cases of a self-detachment of an idiopathic ERM in association with the development of a PVD [1,3,7,11]. This would suggest that the rate of spontaneous release would be higher in idiopathic ERMs that appeared after a PVD had already occurred than those that developed after a PVD occurs (Table 1).

**Table 1:** The rate of epiretinal membrane (ERM) self-detachment. The rate of ERM self-detachment is higher in eyes without a posterior vitreous detachment (PVD) than in eyes after a PVD had occurred.

Report	Duration (month)	The rate of ERM self-detachment (%)	
		PVD at first visit	
		presence	absence
Mayer CH et al., 2004 [8]	2~13	0.47	2.30
Yang H et al., 2014 [7]	33	1.50	13.40

Meyer et al. [8] reported that the self-detachment rate of ERMs was 0.47% in eyes with a preexisting PVD and 2.3% in eyes without a preexisting PVD. These findings were made after two to 13 months of follow-up in their series of 210 patients under the age of 30 years. Yang et al. [7] determined that the incidence of a spontaneous detachment of an ERM was 1.5% in eyes with a preexisting PVD and 13.4% without a preexisting PVD during a 33-month follow-up period of 1248 idiopathic ERM cases. 

Defects of the EZ of the photoreceptors have also been observed in some reports of reduced FMT and improved BCVA after a self-detachment of an idiopathic ERM. These findings suggested that the EZ is susceptible to the vertical tractional forces [1,8], and the ERM recurrence rate was higher in such cases.

In our patient, the FMT decreased to the normal range after the ERM self-detached [10] and an absence of an EZ defect was not observed. This reflects the relatively mild degree of retinal traction and the short duration of membrane adherence [1]. Although recurrences of an ERM are not common, and our case has not had a recurrence and decreased BCVA after 27 months of follow-up, we will continue to monitor the eye for a worsening retinal traction by the residual ERM or a recurrence of the ERM and conduct careful follow-up examinations periodically for a few more years.

Chua et al. [4] reported that 10-30% of idiopathic ERM patients progressed to surgery within a two-to-seven-year period from their first visit. Traditionally, the decision to operate has been based on the severity of symptoms and the effects on visual function as compared with the known surgical morbidity rather than OCT appearance. Indeed, there is a well-known difference between OCT appearance and visual function with some cases of extensive ERM having excellent vision. Several changes in vitreoretinal practice have questioned the traditional practice of surgery only in more advanced cases with reduced vision, and earlier surgery has been advocated. It is known that visual outcomes following surgery are related to preoperative BCVA, and hence early surgery may offer some advantages. However, the visual acuity alone cannot completely represent the visual disabilities due to the condition and other measures of the visual function should be considered. In particular, metamorphopsia and aniseikonia should be prominent symptoms and signs, but their progression over time has not been studied extensively. On the other hand, those who are symptomatic with inner and outer retinal changes are more likely to progress and require surgery. Currently, there are no endpoints that can truly predict which patients would benefit from surgery. Therefore, careful continuous monitoring of the BCVA and the remaining macula thickness in the OCT images, and close communication with the patient on their vision, is needed before the ERM surgery is carried out.

## Conclusions

We have presented our findings of a case of a spontaneous detachment of an idiopathic ERM associated with a PVD with signs of a reduction of traction on the retina. Because a spontaneous detachment of an ERM is rare at 2% to 13%, vitrectomy is usually considered before the macular traction affects the retinal structure. However, in eyes without a PVD, clinicians need to consider the possibility of a development of a PVD and a spontaneous detachment of the ERM. Thus, we recommend that in ERM cases without a preexisting PVD and with good visual acuity and without metamorphopsia, surgery be delayed and careful continuous monitoring for signs of a worsening of the traction on the retina is needed.
